# Anti-NMDA Receptor Encephalitis During Pregnancy: A Case Report

**DOI:** 10.1097/MD.0000000000001034

**Published:** 2015-07-02

**Authors:** Stéphane Mathis, Jean-Christophe Pin, Fabrice Pierre, Jonathan Ciron, Anna Iljicsov, Matthias Lamy, Jean-Philippe Neau

**Affiliations:** From the Department of Neurology, CHU Poitiers, Poitiers (SM, JCP, AI, ML, JPN); Department of Neurology, Hôpital d’Angoulême, Angoulême France (CP); and Department of Gynecology, CHU Poitiers, Poitiers, France (FP).

## Abstract

Anti-*N*-methyl-D-aspartate receptor (anti-MMDAR) encephalitis is an immune-mediated encephalitis mainly affecting young women.

We describe the case of a 21-year-old woman who developed a classical form of anti-NMDAR encephalitis during the 10th week of gestation. The patient had been treated with methylpredinsolone and intravenous immunoglobulins. Birth history of the child was normal, with normal APGAR score. The clinical symptoms of the patient have improved after a few months.

This rare occurrence during pregnancy (only 9 other cases described) presents an opportunity to highlight the importance of making the earliest possible diagnosis of this treatable and potentially reversible encephalitis, and to educate gynecologists, psychiatrists, anesthetists, and neurologists on this potential cause of psychiatric and neurological manifestations during pregnancy.

## INTRODUCTION

In 2005, Vitaliani et al^1^ reported 4 young women who developed acute psychiatric symptoms, seizures, memory deficits, and decreased level of consciousness (often requiring ventilator support), associated with ovarian teratoma. In 2007, Dalmau et al^2^ described 8 additional patients with the same symptoms; these authors finally confirmed the presence of NMDAR (*N*-methyl-D-aspartate receptor) antibodies in all the 12 young women. Anti-NMDAR encephalitis was initially classified as a paraneoplastic syndrome (up to 60% of them are associated with a teratoma or other tumor type), but it is now classified more as an immune-mediated encephalitis.^3,4^ Moreover, since the first clinical descriptions, other cases have been reported in women without teratoma, but also in males and children.^5^ Only a few cases of anti-NMDAR encephalitis were reported to have occurred during pregnancy or postpartum. We report a case diagnosed in the first trimester of pregnancy.

## CASE REPORT

This 21-year-old Caucasian woman developed behavioral changes during the 10th week of pregnancy. She had no medical past history and was first admitted to the Department of Psychiatry for a presumptive depression, she was treated with fluoxetine (and tiapride for agitation), without any improvement. After a few days, because of a worsening of her mental status and then muteness, she was finally admitted to the Department of Neurology where she presented a first generalized seizure.

At that time, the clinical examination showed orofacial and limb dyskinesia, but no pyramidal sign, no motor weakness, no sensory disturbance, no autonomic disturbance, and no abnormality of the cranial nerves; deep tendon reflexes were normal; the body temperature was normal (37.2°C) as well, and we observed no neck stiffness. The first brain MRI (magnetic resonance imaging) was unremarkable. The electroencephalogram showed a generalized slow theta activity without epileptic discharges; anticonvulsivant treatments (clonazepam and lamotrigine) were begun. Results of the cerebrospinal fluid (CSF) analysis showed a lymphocytic pleiocytosis (120 white cells/mm^3^), a moderate increase of the protein level (67 mg/dL; normal value < 45 mg/dL), and a normal glucose level (59 mg/dL). A treatment with acyclovir was started for a presumptive viral encephalitis (also with ampicillin for a few days) but was finally stopped because of the negativity of the polymerase chain reaction herpes simplex and varicella-zoster viruses in the CSF (and the absence of other germs). Other serologies (Epstein-Barr virus, cytomegalovirus, human immunodeficiency virus, *Borrelia burgdorferi*, *Leptospira*, *Coxiella burneti*, and *Mycoplasma pneumonia*) were negative. Other ancillary tests, comprising immunological tests (antinuclear and anti-desoxyribonucleic acid antibodies), were unremarkable; finally, NMDAR antibodies were identified in the CSF 20 days after the first neurological symptoms. An MRI of the abdomen and the pelvis was performed but showed no teratoma or other lesion.

She was treated with methylpredinsolone (3 days, 250 mg/day), without improvement; then, a first course of intravenous immunoglobulins (IVIg) was performed during 5 days (20 g/day), but the patient still presented behavioral disturbances (alternating episodes of catatonia and agitation) and visual hallucinations. One week later, because of a recurrence of seizures, she was admitted in the intensive care unit where she developed a status epilepticus, she gradually lost consciousness, experienced respiratory failure, and was intubated; the symptoms where difficult to control despite treatment with phenytoine, fosphenytoine, and propofol. A second brain MRI showed a diffuse meningeal enhancement (gadolinium) without other lesion, but it was performed only 24 hours after a second lumbar puncture. Two weeks after the first course of IVIg, she received a second course of IVIg (at the same dose). We progressively observed a gradual improvement for the next weeks, but with sequelae: 24 weeks after the onset of the disease, she still presented apathy and episodes of pathological laughing. She gave birth to a healthy girl (weight was 3360 g; APGAR score was 10) who did not present any neurological symptom at 6 months. Nine months after the onset of anti-NMDA receptor encephalitis, cognitive functions of the patient were normal (except for some slight memory disturbance), and anti-NMDA antibodies were negative in her serum.

## DISCUSSION

The lifetime prevalence of mood disorders in women is approximately twice that of men, but this discrepancy (probably in part due to the neuroendocrine events related to female reproduction) is not well understood.^6^ Pregnancy is a time of psychological change and challenge: during the first trimester of pregnancy, a woman may experience increased emotional lability; later, mood disorders (due to bodily changes, alterations in sexual interest, and anxieties about delivery) may appear; late pregnancy may be associated with social withdrawal and increased absorption and preoccupation with preparations for delivery and caring of the baby, sometimes with obsessive thoughts focusing on the health of the baby.^7,8^ Pregnancy and the postpartum period are known to influence the onset and course of various psychiatric disorders: 10% of gravid women meet criteria for major depression, and up to 18% of women show elevated depressive symptomatology during gestation; postpartum depression is also a common disorder occurring in 15% of deliveries.^9^ However, during pregnancy, neurological disorders, such as “encephalopathy,” may also represent a potential cause of psychiatric troubles.

Anti-NMDAR encephalitis is a rare and severe immune-mediated disease usually affecting young female of reproductive age with ovarian tumors (teratoma); however, it was also observed in female of all ages (with or without neoplasm), male, and children (children under 12 and male patients rarely having a tumor).^5^ Only 10 cases,^10–17^ comprising our case (the case of Ito et al was reported in the series of 3 cases of Kumar et al), have been reported in young women during pregnancy (Table 1  ); one of these patient**s** presented a concomitant autoimmune Graves’ hyperthyroidism and anti-NMDAR encephalitis.^16^ Two other cases were reported during the postpartum period,^18,19^ and 1 other paper described a woman with known anti-NMDAR encephalitis (5 years before) who become pregnant.^20^ The clinical presentation of anti-NMDAR encephalitis is usually complex: one of its characteristics is the presence of psychiatric manifestations that meet criteria for the diagnosis of delirium due to a general medical condition (GMC), psychotic disorder due to a GMC and catatonic disorder due to a GMC (where GMC is NMDAR encephalitis), which is a reason why some of these patients are first admitted in a Department of Psychiatry.^21^ In order to make the earliest possible diagnosis, it is crucial to seek the other signs of this immune-mediated encephalitis.

**TABLE 1:**
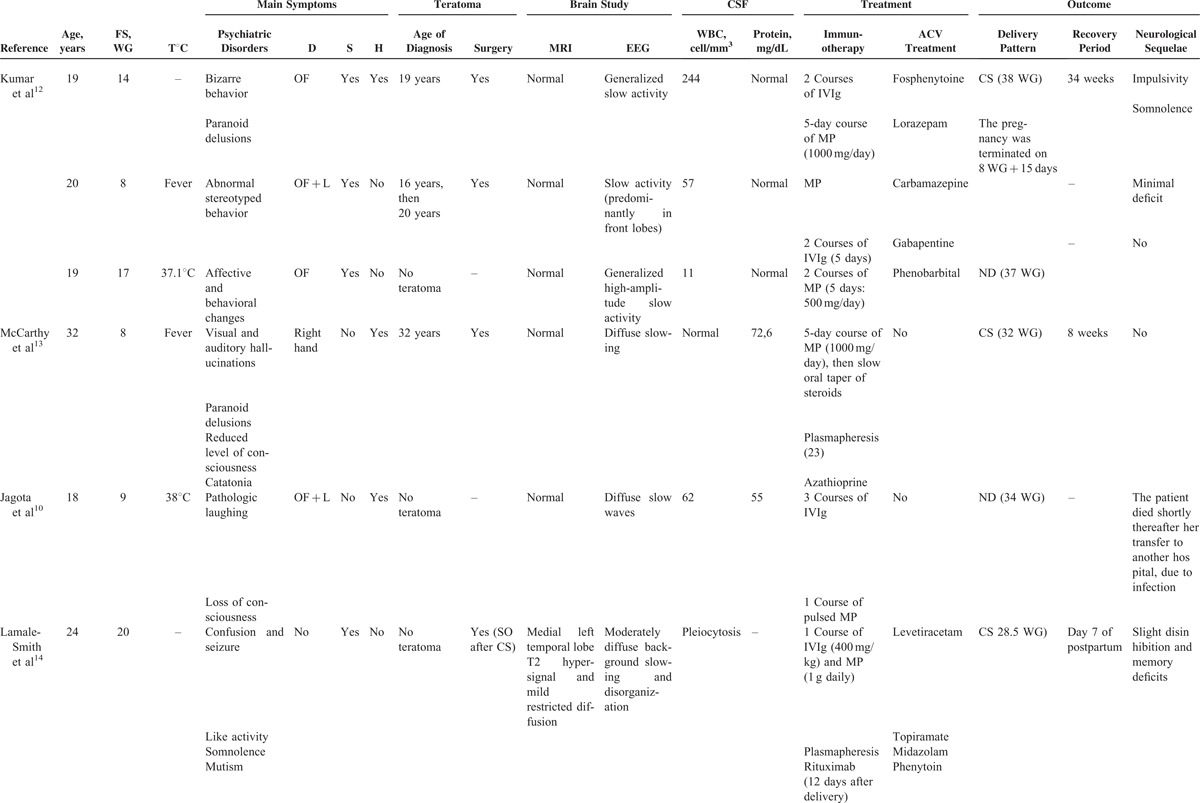
Main Clinical, Biological, and Radiological Data of Patients With Anti-NMDA Receptor Encephalitis During Pregnancy

**TABLE 1 (Continued):**
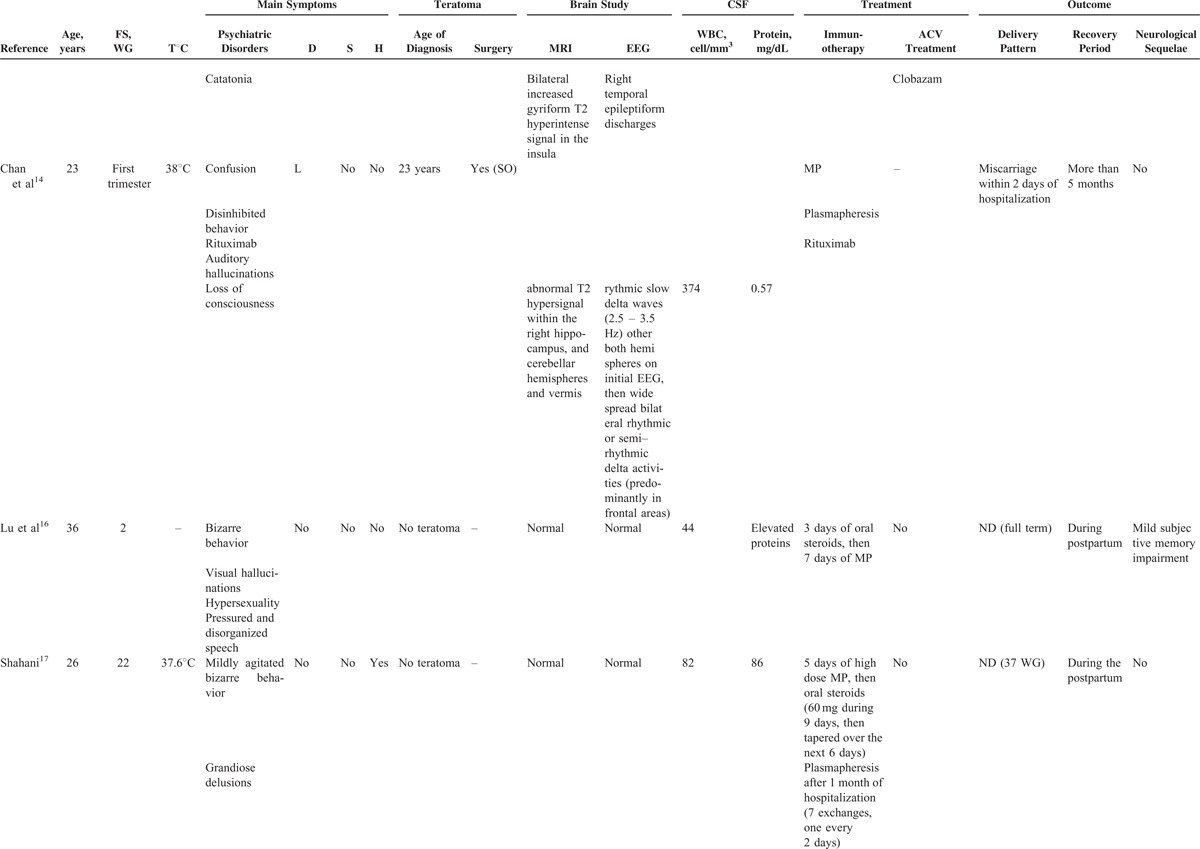
Main Clinical, Biological, and Radiological Data of Patients With Anti-NMDA Receptor Encephalitis During Pregnancy

**TABLE 1 (Continued):**
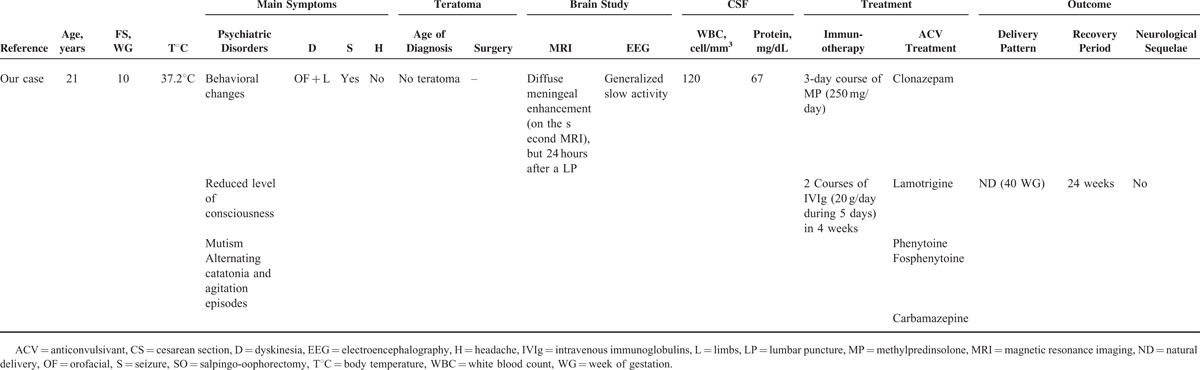
Main Clinical, Biological, and Radiological Data of Patients With Anti-NMDA Receptor Encephalitis During Pregnancy

In 70% of patients, the clinical course begins with viral prodromes (headache, fever, malaise, inability to concentrate, nausea, diarrhea, and vomiting) lasting 5 to 14 days, followed by a seizure and/or psychotic phase characterized by emotional and behavioral disturbances such as apathy, fear, depression, decreased cognitive skills, and psychosis (delusions and hallucinations); sometimes, choreiform movements and ataxia are observed. Patients then present an unresponsive phase (sometimes with muteness and akinesia) and a hyperkinetic phase comprising dyskinesias (and other stereotyped motor automatisms) and autonomic instability (cardiac arrhythmia, hypotension, hypertension, hyperthermia, hypothermia, and hypoventilation) usually requiring intensive care unit-level.^21^ About half of the patients show MRI irregularities, commonly T2 or FLAIR hyperintensities in cortical and subcortical brain region (sometimes with mild or transient contrast enhancement).^22^ In most cases, electroencephalography is abnormal with slow and disorganized activity in the delta/theta range (sometimes with superimposed electrographic seizures).^22^ CSF findings include moderate lymphocytic pleiocytosis, elevated protein level, and oligoclonal bands in 60% of cases.^23^ Final diagnosis is based on the presence of IgG antibodies in the GluN1 (NR1) subunit of the NMDAR in the serum or CSF.^3^ The use of early and aggressive immunosuppressive treatment (corticosteroids, IVIg, and plasma exchange), along with removal of tumor (if present), leads to positive outcome in 80% of patients (returning to nearly basal level of function),^5^ mortality is estimated to be 4%.^24^ Because anti-NMDAR encephalitis is a treatable and potentially reversible encephalopathy, some authors suggest systematically considering this quite novel diagnosis in patients with “encephalitis of unknown origin,” “drug-induced psychosis,” and “new onset epilepsy.”^25^

Among the 10 women who have presented an anti-NMDAR encephalitis during pregnancy, 2 pregnancies were terminated during the first trimester; among the 8 other cases, only 4 newborns were tested for the antibodies in the umbilical cord blood, serum, or CSF: 2 were negative,^12,17^ but the titer of NMDAR antibodies was at the same level as the mother's at birth for the 2 others (then declined and was negative).^10,15^ In one of these cases of maternal transfer of NMDAR antibodies, the baby presented cortical dysplasia and developmental delay (3 years of follow-up), but without confirmation of a link between NMDAR antibodies and the brain abnormalities,^10^ the authors having used a variant of the test that is not specific, as suggested in a recent report by the same group (showing lack of specificity for anti-NMDAR encephalitis in 23.2% of the patients).^26^

All the other babies (as in our observation) were reported to be normal, but with a maximal follow-up of 6 months. This question of a transplantal transfer of NMDAR antibodies from mother to child appears to be crucial because of the ability of NMDAR antibodies to alter the density and synaptic localization of NMDAR in animal models.^27^ The NMDA receptors also have an important role in the brain development, and a glutamatergic dysfunction (particularly with involvement of NMDAR) plays an important role in the genesis of schizophrenia.^5^ Long-term follow-up of the children with mothers who developed NMDAR antibodies during pregnancy should give us more certainty.

## CONCLUSIONS

Behavioral changes occurring during pregnancy may be the consequence of neurologic diseases such as anti-NMDAR encephalitis. It is of the utmost importance for gynecologists, psychiatrists, anesthetists, and neurologists to be aware of this treatable and potentially reversible immune-mediated disorder, and to provide an early diagnosis and prompt treatment.
